# Standardized *Astragalus Mongholicus* Bunge-*Curcuma Aromatica* Salisb. Extract Efficiently Suppresses Colon Cancer Progression Through Gut Microbiota Modification in CT26-Bearing Mice

**DOI:** 10.3389/fphar.2021.714322

**Published:** 2021-08-31

**Authors:** Junfei Gu, Ruolan Sun, Qiaohan Wang, Fuyan Liu, Decai Tang, Xiangwei Chang

**Affiliations:** ^1^ School of Traditional Chinese Medicine and School of Integrated Chinese and Western Medicine, Nanjing University of Chinese Medicine, Nanjing, China; ^2^ School of Pharmacy, Anhui University of Chinese Medicine, Hefei, China

**Keywords:** colon cancer, gut microbiota, astragalus mongholicus bunge-curcuma aromatica salisb., intestinal barrier, short-chain fatty acids, SDF-1/CXCR4

## Abstract

Altered gut microbiota and a damaged colon mucosal barrier have been implicated in the development of colon cancer. *Astragalus mongholicus* Bunge-*Curcuma aromatica* Salisb. (ACE) is a common herbal drug pair that widely used clinically to treat cancer. However, whether the anti-cancer effect of ACE is related to gut microbiota remains unclear yet. We standardized ACE and investigated the effects of ACE on tumour suppression and analyze the related mechanisms on gut microbiota in CT26 colon cancer-bearing mice in the present study. Firstly, four flavonoids (calycosin-7-glucoside, ononin, calycosin, formononetin) and three astragalosides (astragaloside A, astragaloside II, astragaloside I) riched in *Astragalus mongholicus* Bunge, three curcumins (bisdemethoxycurcumin, demethoxycurcumin, curcumin) and four essential oils (curdione, curzerene, germacrone and β-elemene) from *Curcuma aromatica* Salisb., in concentrations from 0.08 to 2.07 mg/g, were examined in ACE. Then the results *in vivo* studies indicated that ACE inhibited solid tumours, liver and spleen metastases of colon cancer while simultaneously reducing pathological tissue damage. Additionally, ACE regulated gut microbiota dysbiosis and the short chain fatty acid content in the gut, repaired intestinal barrier damage. ACE treatment suppressed the overgrowth of conditional pathogenic gut bacteria, including *Escherichia-Shigella*, *Streptococcus* and *Enterococcus*, while the probiotic gut microbiota like *Lactobacillus*, *Roseburia*, Prevotellaceae*_UCG-001* and *Mucispirillum* were increased. More interestingly, the content level of SCFAs such as propionic acid and butyric acid was increased after ACE administration, which further mediates intestinal SDF-1/CXCR4 signalling pathway to repair the integrity of the intestinal barrier, decrease Cyclin D1 and C-myc expressions, eventually suppress the tumor the growth and metastasis of colon cancer. To sum up, the present study demonstrated that ACE could efficiently suppress colon cancer progression through gut microbiota modification, which may provide a new explanation of the mechanism of ACE against colon cancer.

## Introduction

Colorectal cancer (CRC) is reported to be a leading cause of death worldwide, the mean age at diagnosis is getting younger, and the incidence of colon cancer is higher than that of rectal cancer (Siegel et al., 2020). Local recurrence and distal metastasis rates after surgical resection contribute to the lethal nature of CRC (Backes et al., 2019). Effective therapies for the elimination of solid tumours, disseminated metastatic nodules and the simultaneous prevention of tumour recurrence are urgently needed. It is not surprising that the intestinal microbiome, the barrier that mediates between our environment and our genes, has been seen as a main cause of many diseases, such as colon cancer, diabetes, obesity, and the development of autoimmune diseases (Imhann et al., 2018; Sethi et al., 2018; Olsson et al., 2020). Direct or indirect interventions to regulate disease progression by affecting the intestinal microbiome, through strategies such as faecal transplantation, drugs and diet, have become a hot new topic of research (Bauer et al., 2018; Huang F. et al., 2019; Pop et al., 2020).

Symbiotic bacteria can directly contribute to the development of colon cancer by increasing gene mutations in epithelial cells or activating specific oncogenic pathways, while the flora can strongly regulate the host’s immune system to influence tumour growth indirectly (Cremonesi et al., 2018). Single species with oncogenes have been identified as a result of developments in biological technology, including BFT toxin expressed by enterotoxigenic *Bacteroides fragilis* (ETBF), pks virulence islands by *Escherichia coli*, and the FadA and Fap2 adhesins by *Fusobacterium nucleatum* (Arthur et al., 2012; Bullman et al., 2017).

Traditional Chinese medicine (TCM), the largest application category including Chinese herbal medicine (CHM), has been widely used as a main complementary and alternative therapy for cancer patients because of its effectiveness and few side effects (Zhai et al., 2018; Huang WC. et al., 2019; Peng et al., 2019; Chan et al., 2021). In previous studies, we found that the mixed preparation of extract of *Astragalus mongholicus* Bunge [Fabaceae; Astragali Radix]- *Curcuma aromatica* Salisb. [Zingiberaceae; Curcumae Rhizoma] (ACE) produced synergistic effects on promoting the extract of bioactive ingredients, such as curcumenol, curdione, isocurcumenol, furanodienone, curcumol, and germacrone (Yin et al., 2018). And the ACE intervention showed a good effect on lung, liver, and colon cancers and was particularly effective in inhibiting liver metastases from colon cancer (Tang et al., 2019; Sun et al., 2021). However, whether the inhibitory effect is related to gut microbiota regulation is still not clear.

In this study, we sought to investigate the gut microbiota that occurs in the setting of colon cancer, which might prompt tumour formation and distal metastasis. We hypothesized that ACE-induced alterations in the gut microbiota inhibit the development and metastasis of colon cancer through certain pathways. Targeting such mechanisms may provide a new way to develop therapeutics against colon cancer and liver metastasis, and provide ideas for the development of antitumour Chinese medicine like *Astragalus mongholicus* Bunge-*Curcuma aromatica* Salisb.**

## Materials and Methods

### Sample and Standards Preparation

Sample of *Astragalus mongholicus* Bunge [Fabaceae] and *Curcuma aromatica* Salisb. [Zingiberaceae], authenticated by Professor Tulin Lu of Nanjing University of Chinese Medicine, used in the study were prepared as before (Sun et al., 2021). 400 g *Astragalus mongholicus* Bunge and 200 g *Curcuma aromatica* Salisb. (at the optimized proportion of 2:1) were weighted and extracted two times with 10 volumes of water for 60 min each time using a heat reflux system. Meanwhile, the essential oil components were recovered via a Soxhlet extract unit. The extraction was freeze-dried to powder after coalescing the twice filtrates, and the extraction rate was 11.71%. The freeze-dried extract powder and essential oils were preserved in the Key Laboratory of High Technology of Prescription Research in Jiangsu Province (NO. 20190654-AC).

The *Astragalus mongholicus* Bunge standards astragaloside Ⅰ (C16J8G37958), astragaloside II (YM0306HD14), astragaloside (J04M8T30363), formononetin (F27J7S18516), ononin (R28O8F46957), calycosin (Y24N9Y75652) and calycosin-7-glucoside (Y27F9H54731) were all provided by Shanghai Yuanye Biotechnology Co. For the standards of *Curcuma aromatica* Salisb., β-elemene (100268-201903, China Academy of Food and Drug Administration, Beijing, China), curcumene (JBZ-0330, Nanjing Jin Yi Bai Biotechnology Co., Ltd., Beijing, China), curdione (LW17090612), germacrone (LW17040709), curcumin (LW17091410), demethoxycurcumin (LW16090803) and bis-demethoxycurcumin (LW16090905), the purity of each standard was over 98%, and all these standards were purchased from Nanjing Liangwei Biotechnology Co.

### UPLC–MS/MS Analysis Conditions

An ACQUITY UPLC™ (Waters, Milford, MA, United States) system equiped with a Q-Trap^®^ 6500^+^, a triple quadrupole/composite linear ion trap mass spectrometer with a Turbo V™ ion source, an Analyst V1.6.3 workstation and MultiQuant™ data processing software (AB SCIEX, CA, United States) was used for the quantitative analysis. An ACQUITY™ UPLC BEH C18 (100 mm × 2.1 mm, 1.7 μm) column was used. The column was operated with column temperature of 30°C, flow rate of 0.4 ml/min, autosampler temperature of 10°C and injection volume of 2 μl. The mobile phase included phase A (water with 0.1% formic acid) and phase B (acetonitrile), and linear gradient elution was performed as below: 5-20% B, 0-2 min; 20-45% B, 2-6 min; 45-70% B, 6-12 min; 70-80% B,12-13 min; 13-14 min, 80-100% B. All samples were dissolved in 80% methanol in water, and the supernatant was extracted as the test solution after a 0.22 μm microporous membrane filtering.

The MS/MS parameters were optimized as follows: positive and negative modes; ion-spray voltages: 4.5 and 5.5 kV; curtain gas: 35 psi; nebulizer gas (GS1, nitrogen): 45 psi; auxiliary heater gas (GS2, nitrogen): 50 psi; ion source atomization temperature: 400°C; scanning method: multi-reaction ion monitoring (MRM).

### Animal Modelling

Cell culture: CT26. WT cells were obtained from Beijing Cancer Research Institute. RPMI-1640 medium with 10% foetal bovine serum, penicillin (100 U/ml), and streptomycin (100 U/ml) were chose to culture the cells. And cells were incubated in a humidified atmosphere at 37°C and 5% CO_2_.

Orthotopic transplantation model of colon cancer and grouping: The colon cancer mice was modelled as the orthotopic transplantation tumour model according to previous operation (Jackstadt and Sansom, 2016; Sun et al., 2021). Male BALB/c mice (3-5 weeks, weight 20 ± 2 g) were kept in the Experimental Animal Center of Nanjing University of Chinese Medicine (Nanjing, Jiangsu, China) at in a constant atmosphere at 26°C temperature and 45% humidity. All mice were provided by Huaxing Experimental Animal Farm (Zhengzhou, Henan, China). The experimental animal licence number was SCXK (Yu) 2019-0002. Five breeder mice were vaccinated with 1×10^6^ CT26 cells in the right axilla. Then, the mice were sacrificed about 5 days until the tumor in the axilla grows to a soybean size. The tumor was peeled off and the fish-like cancer tissue was cut into small cubes of 1 mm^3^.

After isoflurane anesthesia, the left lower abdome of model mice were incised. Then tumour cube was adhered to the scratched caecum site with histoacryl adhesive in model mice. The wound was sewed and treated with anti-infective operation, and the mice were returned to cages. The success rate of the orthotopic-transplanted colon cancer model was 98.18%. The sham group underwent all operations except for tumour grafting. The 50 cancer-bearing mice were randomly divided into five groups: the model group; the 5-FU group (30 mg/kg/3 days); ACE_L group (0.32 g/kg/day); ACE_M group (0.64 g/kg/day); and ACE_H group (1.28 g/kg/day). 10 mice in each group received drug administration for 15 days. Mice in the sham and model groups were administered normal saline at the same dose. Except for the intraperitoneal injection of 5-FU every 3 days, the other intervention agents were given by intragastric administration once a day.

The entire operation process followed the principle of aseptic operation and was in compliance with the National Institute of Health Guide for the Care and Use of Laboratory Animals, and no antibiotics are required after surgery.

### Sample Collection

Stool specimen collection: Mice were fixed, and the tails were lifted. The lower abdomen was stroked with fingers to promote defecation. Fresh faeces were collected with sterile EP tubes, placed in liquid nitrogen, then transferred to the refrigerator (−80°C) for storage.

Orbital blood serum: Mouse blood was obtained via the orbital vein. The supernatant was collected as the serum after 1,500 × g centrifugal speed for 10 min after drug treatment, and stored at −80°C.

Tissue sample collection: An abdominal autopsy was performed The intact liver, spleen and solid tumours were collected out and weighed after mice sacrificed with cervical dislocation, and the number of metastases in the liver and spleen were observed and recorded. The collected tissue samples were placed in 4% paraformaldehyde or liquid nitrogen.

### Genomic DNA Extract, 16S Amplification and Illumina Sequencing

The gut microbiota sequencing assay was consistent with our previous study (Gu et al., 2017). 16S amplification DNA were quantified by a PowerSoil DNA isolation kit (MO BIO Laboratories, Carlsbad, CA, United States) and NanoDrop 2000 Spectrophotometer (Thermo, Waltham, United States). PCR primers for 16S rRNA amplification were used with 319F and 806R to amplify the hypervariable V3-V4 region. Then, amplicon pyrosequencing was performed on an Illumina MiSeq^®^ system (Illumina, Inc., San Diego, CA, United States) according to the manufacturer’s instructions at LC Biotech Co., Ltd., Hangzhou, Zhejiang, China. The raw data of libraries generated during this study is publicly available at the Sequence Read Archive (SRA) portal of NCBI (https://www.ncbi.nlm.nih.gov/sra/) under accession number PRJNA738850.

Sequence data were analysed by FastQC (Version 0.10.1) and QIIME software (Version MacQIIME 1.9.1-20150604). A 97% high-quality 16S rRNA sequence identity were clustered into operational taxonomic units (OTUs) with via CD-HIT (Version 4.6.8). The taxonomy of all high-quality sequences was selected to recalculate and clustered by the UniFrac method with the R software package. The alpha_diversity.py script, computed by rarefaction of OTUs, was used to calculate metrics such as observed OTUs, Shannon index, Simpson index, and Chao1 index. The beta_diversity_through_plots.py script was also run to visualize beta diversity.

### Gas Chromatography Mass Spectrometry

To determine the targeted fatty acid quantitation, faecal samples from the mice were harvested, homogenized, and snap frozen in liquid N_2_. The standards (acetic acid, propionic acid, butyric acid, isobutyric acid, valeric acid, isovaleric acid, and hexanoic acid, purity ≥99.0%) provided by Sigma-Aldrich Corp (St. Louis, MO, United States) were thawed on ice, and ddH_2_O 300 μL, isopropanol/pyridine solution (500 μL, 3:2, v/v), and PCF solution 100 μL were used to the derivatization reaction. The experimental conditions and calibration curves for SCFA quantification were optimized by the derivatized standards, and 50 μL DL-2-methylbutyric acid was added as an internal standard. Approximately 30 mg of faecal sample was weighed and homogenized with 15% phosphoric acid 50 μL, 125 μg/ml internal standard 100 μL and ether 400 μL for 1 min. The supernatant was obtained for SCFA detection after centrifuging at 5,000 × g for 10 min at 4°C.

A Thermo Scientific TRACE™ 1310-ISQ LT GC-Ion Trap MS instrument coupled with a TRACE™ 1310 gas chromatography system and a 1310 autosampler (Thermo Fisher Scientific, Waltham, MA, United States) was used to detect SCFAs. An Agilent HP-INNOWAX capillary column (30 m × 0.25 mm ID × 0.25 μm, Santa Clara, CA, United States) was used for SCFA separation. The sample was injected in a split flow with 1 μL injection at a ratio of 10:1. The ion source temperature was 230°C, the transmission line was 250°C and the quadrupole temperature was 150°C. The programme was started at 90°C, followed by 120°C at 10°C/min, 150°C at 5°C/min and 250°C at 25°C/min for 2 min. The carrier helium gas was at 1.0 ml/min. The energy of electron ionization (EI) was 70 eV. As shown in the TIC diagram (Supplementary Figure S1), all short-chain fatty acids can be distinguished.

### Histopathological Studies

The colon tissues and solid and liver tumours were dehydrated and embedded in paraffin. 3 mm paraffin specimens were rehydrated and stained with haematoxylin and eosin (H&E). Histological images were observed with an IX51 microscope (Olympus Corporation, Japan).

### Enzyme-Linked Immunosorbent Assay

The contents of zonulin (H389-1-2), IL-6 (H007-1-2), LPS (H255-1-2), IL-22 (H019-1-2) and IL-17A (H014-2-2) in serum were detected using ELISAs. All assay kits were obtained from Nanjing Jiancheng Bioengineering Institute (Nanjing, Jiangsu, China). Related experiments were perated and calculated according to the instructions.

### Western Blot Analysis

Proteins were extracted from solid tumours and colon tissues with 1% phosphatase inhibitor cocktail, quantified by BCA protein assay kit (Thermo Fisher Scientific, Waltham, MA, United States). 10% SDS–PAGE were chose to resolve samples and then transferred to PVDF membranes (EMD Millipore, Burlington, United States). After blocking with 5% BSA for 2 h, the membrane was incubated with primary antibodies: occludin (1:1000; ab167161, Abcam, Cambridge, United Kingdom), SDF-1 (1:1000; ab9797, Abcam), CXCR4 (1:1000; ab124824, Abcam), ZO-1 (1:1000; CST#13663, Cell Signaling Technology, Darmstadt, Germany), cyclin D1 (1:500; CST#2978, Cell Signaling Technology) and c-myc (1:500; CST#5605, Cell Signaling Technology), then gentle shaked overnight at 4°C. After washing, the membranes were incubated with the second antibodies for 1 h (goat anti-rabbit or IgG anti-mouse, 1:5,000; Cell Signalling Technology). Immunoreactivity was visualized with an enhanced chemiluminescence system (ECL kit; Santa Cruz Biotechnology, United States) and quantified using Image Lab 4.0 (Bio-Rad, Hercules, CA, United States).

### Statistical Analysis

The datas are calculated as the mean ± standard error (mean ± SE), and SPSS software (version 22.0, IBM Corp, Chicago, IL, United States) were used to performe statistical analyses. The independent samples *t* test was used to compare normally distributed data with homogeneous variances between two groups. The Mann-Whitney nonparametric test was used when there was no assumption of a normal distribution. Analysis of Variance (ANOVA) was used to calculate the significant differences in basal characteristics between the groups. *p* values < 0.05 were considered significant.

## Results

### Standardization of ACE

**FIGURE 1:**
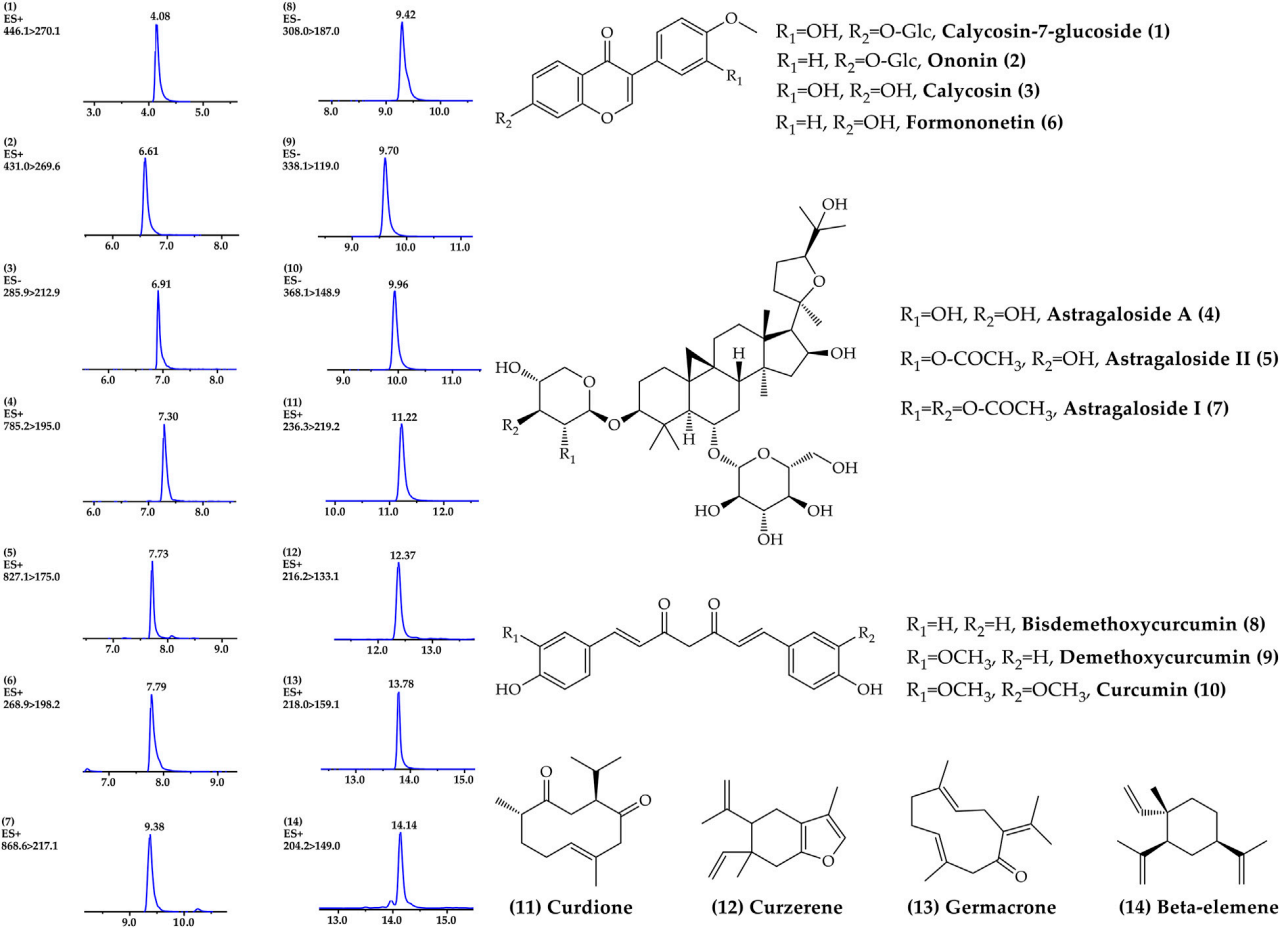
Chemical profile of *Astragalus mongholicus* Bunge-*Curcuma aromatica* Salisb. extract detected by UPLC–MS/MS.

A rapid, sensitive and accurate UPLC-MS/MS analysis method was established to reveal the chemical profile and quantify the main ingredients of ACE extract before drug treatment. 14 compounds, including calycosin-7-glucoside 1), ononin 2), calycosin 3), astragaloside A 4), astragaloside II 5), formononetin 6), astragaloside I 7), bisdemethoxycurcumin 8), demethoxycurcumin 9), curcumin 10), curdione 11), curzerene 12), germacrone 13) and β-elemene 14) were identified from ACE through the individual standards comparison (Figure 1, Supplementary Table S1). Their contents were 1.15, 0.13, 0.30, 1.19, 0.18, 0.09, 0.63, 0.08, 0.17, 0.57, 0.73, 0.67, 0.42 and 2.07 mg/g, respectively. The main ingredients analysis results served as a quality control for the reproducibility of the follow-up research.

### ACE Inhibited Colon Cancer Tumour Growth in Tumour-Bearing Mice

**FIGURE 2:**
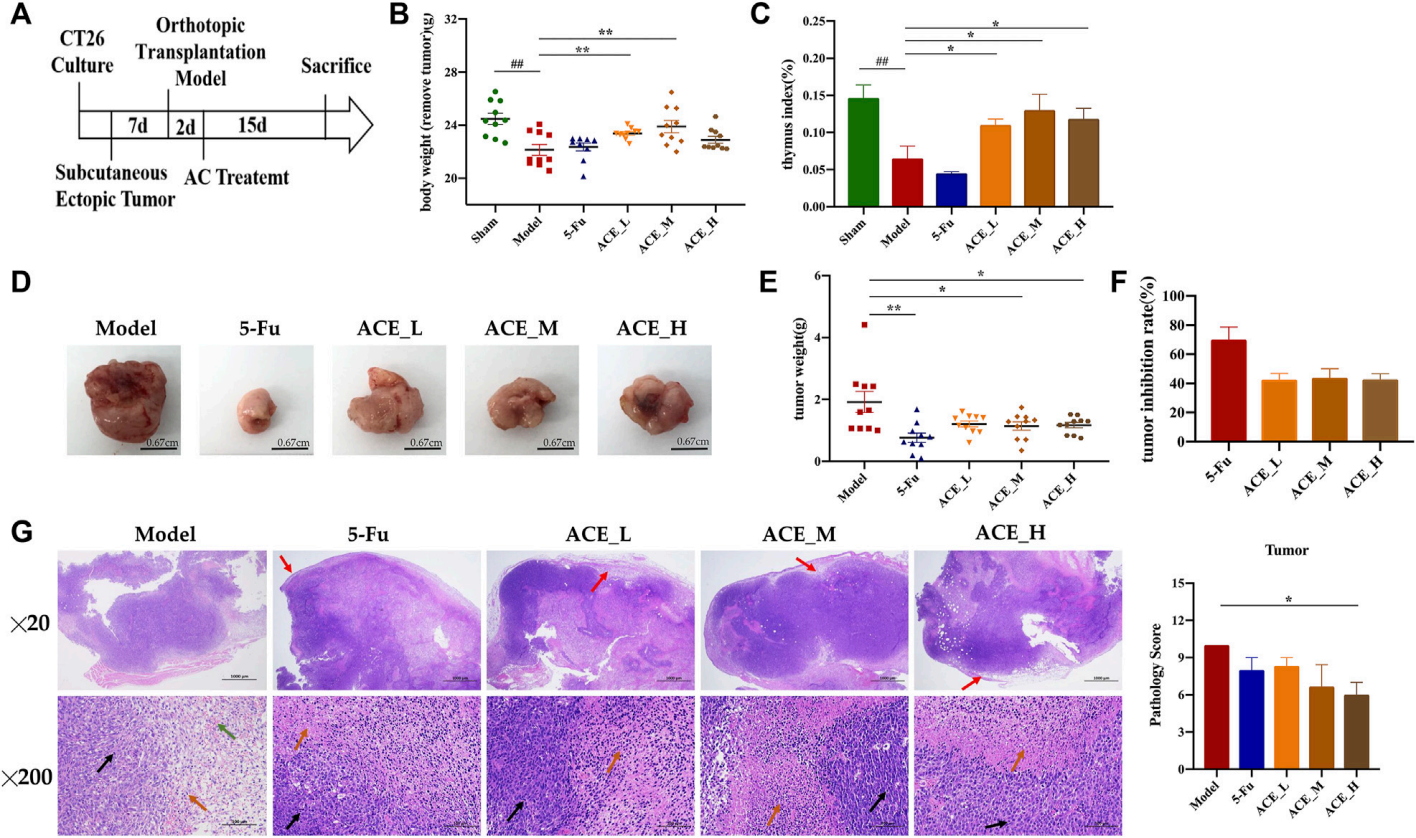
The effect of *Astragalus mongholicus* Bunge-*Curcuma aromatica* Salisb. extract on solid tumours of colon cancer-bearing mice. **(A)** Schematic diagram of the experimental design process; **(B)** Body weight after tumour removal of mice after ACE administration; **(C)** Thymus index of mice after ACE administration; **(D)** Pictures of solid tumours in each group; **(E)** Tumour weight; **(F)** Tumour suppression rate; **(G)** Tumour histopathological changes (haematoxylin and eosin (HE) stain). Inflammatory cells (red arrows), disordered nuclei (black arrows), haemorrhage with red blood cells (green arrows) in tumour cells, cytoplasmic eosinophilia (orange arrows). ^##^ *p* < 0.01, compared with the sham group; ^*^ *p* < 0.05 and **p < 0.01, compared with the model group. n = 10.

During the course of administration (Figure 2A), the mice in the sham-operated group had soft and healthy fur and were active. In contrast, the mice in the model group had increasingly bulging abdomens, relatively grey fur, were less active and less responsive to stimuli. Compared with the mice in the model group, mice treated with ACE had relatively healthy fur and were more active while the mice in the 5-FU group were less active. Body weight after tumour removal and thymus indices of the mice in the model and 5-FU groups were significantly lower than the sham mice (*p* < 0.01), while the mice in the ACE group had significantly higher body weight (without tumour) and enhanced immune function compared with tumour-bearing mice in model group (*p* < 0.01, Figures 2B,C). After administration, the tumour weights and sizes of colon cancer tumours were suppressed in all drug-treated mice (Figures 2D–F). After 5-FU administration, the tumour weight of cancer-bearing mice was decreased significantly (*p* < 0.01, Figure 2D), and the tumour inhibition rate was the highest among all groups, at 65.92% (*p* < 0.01, Figure 2F). Tumour growth was also significantly inhibited by ACE administration (*p* < 0.05), and the medium dose showed a better tumour suppression rate than the high and low doses, with a rate of 43.43%. In the sham group, the junctional tissue of the caecum, the same location as the tumour implantation site of the model mice, was taken as a negative control. The intestinal mucosal epithelium was intact, the intestinal glands were abundant and closely arranged, and the number of cupped cells was abundant. In the model group, the HE staining of cancer tissues showed obvious inflammatory cell infiltration (red arrows), disordered nuclei (black arrows), and haemorrhage with red blood cells (green arrows) alongside tumour cells. The nuclei were stained, fragmented and lysed, with increased cytoplasmic eosinophilia (orange arrows). Tumour cells in the 5-FU group were seen to have large areas of tissue necrosis. After ACE administration, the alteration tumour nuclear staining was less than model cancer-bearing mice, and the pathological score was significantly improved (*p* < 0.01, Figure 2G). These results demonstrated that ACE could improve the body weight of tumour-bearing mice and inhibit the growth of colon cancer to a certain degree.

### ACE Reduced Liver Metastases in Tumour-Bearing Mice

**FIGURE 3:**
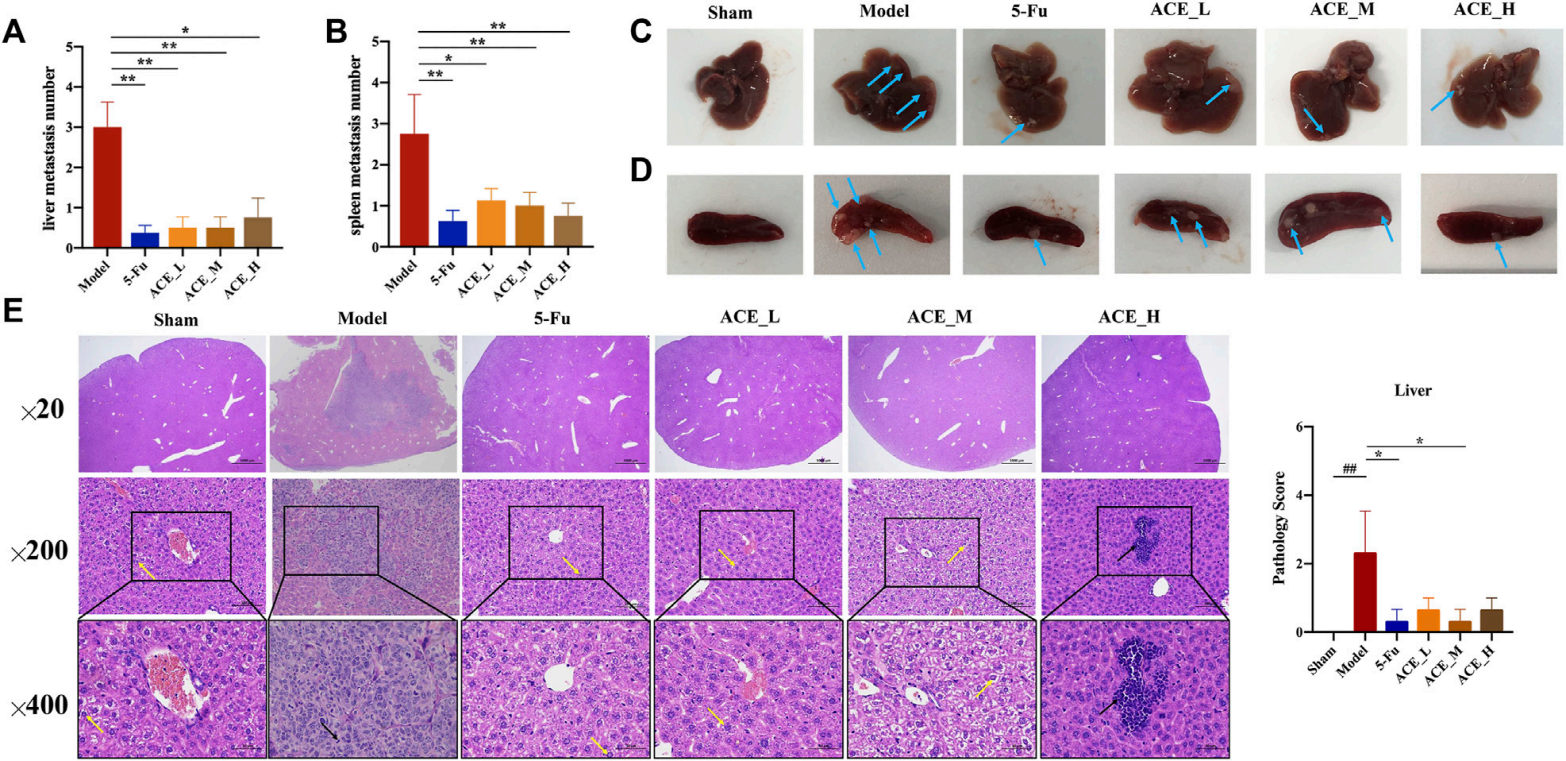
The effect of *Astragalus mongholicus* Bunge-*Curcuma aromatica* Salisb. extract on metastases of colon cancer-bearing mice. **(A)** Liver metastasis number of mice after ACE administration; **(B)** Spleen metastasis mumber of mice after ACE administration; **(C)** Pictures of liver metastasis nodules in each group (blue arrows); **(D)** Pictures of spleen metastasis nodules in each group (blue arrows); **(E)** Liver metastasis histopathological changes (haematoxylin and eosin (HE) stain). Granular cytoplasm (yellow arrows) and altered nuclear staining (black arrows). ^##^ *p* < 0.01, compared with the sham group; ^*^ *p* < 0.05, compared with the model group. n = 10.

In the morphological analysis of liver tissues after dissection, obvious metastatic foci were seen on the liver and spleen of mice in the model group as single or multiple white nodules. The number of liver metastatic nodules was lower than that in the model group after drug intervention (blue arrows, Figures 3A–D). Compared with those of the sham group, the livers of colon cancer-bearing mice exhibited hepatocyte granular degeneration with loose, lightly stained granular cytoplasm (yellow arrows) and altered nuclear staining (black arrows). The histopathological changes in the liver in all herbal treatment groups were improved (*p* < 0.01, Figure 3E). Therefore, the ACE combination for colon cancer could reduce the number of liver metastases and inhibit liver metastasis in mice with colon cancer.

### ACE Modulated the Gut Microbiota

**FIGURE 4:**
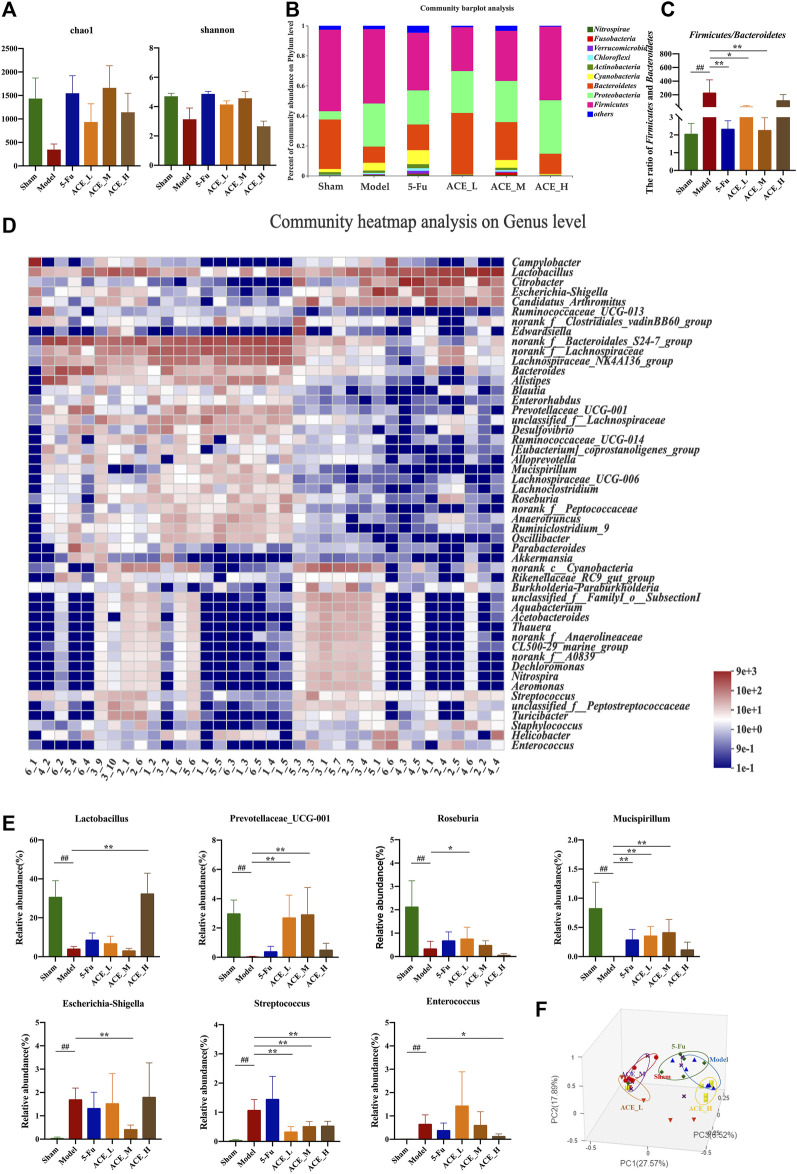
The effect of *Astragalus mongholicus* Bunge-*Curcuma aromatica* Salisb. extract on the gut microbiota of colon cancer-bearing mice. **(A)** Alpha diversity index analysis (Chao and Shannon indices); **(B)** Community bar plot analysis of gut microbiota at the phylum level; **(C)** The ratio of relative abundances of Firmicutes and Bacteroidetes; **(D)** Community heatmap analysis at the genus level; **(E)** Representative differentiated genera; **(F)** PCoA analysis of gut microbiota. ^##^ *p* < 0.01, compared with the sham group; ^*^ *p* < 0.05 and **p < 0.01, compared with the model group. n = 6.

The sequencing data reached saturation and were able to cover the majority of species in the mouse gut microbiome community (Supplementary Figure S2). The results of the microbial community alpha diversity index showed differences in diversity between individual samples. The results from the Chao and Shannon indices revealed that the alpha diversity of mouse faeces after modelling was lower than that in the sham group, implying that the abundance and diversity of the gut microbiota decreased after colon cancer modelling. The intestinal microbial diversity and richness of colon cancer mice were improved to different degrees after intervention treatment with ACE, especially in the middle dose group (Figure 4A, Supplementary Figure S1).

At the phylum level, as shown in Figure 4B, the dominant microbiota phyla were *Firmicutes* and *Bacteroidetes*. Compared with those in the sham mice, the relative abundances of *Proteobacteria* and *Cyanobacteria* were increased, while the abundances of *Firmicutes* and *Bacteroidetes* were decreased, and the structure of the flora was changed. The change in the relative abundance of microbiota at the phylum level was reversed after ACE administration. The ratio of *Firmicutes* and *Bacteroidetes* in modelling drug-free mice was significantly increased compared with that in the sham group (*p* < 0.01, Figure 4C). The ratio was decreased after ACE intervention, and the difference was significant in the medium dose (0.64 g/kg) group compared with the model group (*p* < 0.05). At the genus level, significant differences were shown in the composition and abundances of intestinal flora in the model group compared to the normal group. Compared to that of the sham group, the microbiota structure of the model group was significantly altered (Figures 4A–D). Among the 21 top differentially abundant genera (Supplementary Figure S3), the abundances of *Escherichia-Shigella*, *Streptococcus* and *Enterococcus* were increased significantly, while those of *Lactobacillus*, Prevotellaceae*_UCG-001, Roseburia* and *Mucispirillum* were significantly reduced (*p* < 0.01, Figure 4E). The ACE intervention significantly restored the levels of these genera (*p* < 0.05, *p* < 0.01). However, 5-FU had a smaller effect on the reversion of intestinal genus levels in colon cancer-bearing mice, and it even tended to increase further for *Streptococcus*. Principal coordinate analysis (PCoA) revealed similarities and differences between samples and groups. The results of the analysis showed that the samples in the model group were far apart from those in the sham group, indicating successful modelling. The majority of the samples in the ACE administration group were distributed between the model and sham groups; the samples from the ACE_L and ACE_M groups were close to those from the sham-operated group, while the samples from the ACE_H and 5-FU groups were close to those from the model group (Figure 4F). Therefore, ACE was able to modulate the abundances of intestinal flora members and regulate the intestinal flora structure in colon cancer model mice.

### ACE Improved Changes in SCFAs and Intestinal Barrier Integrity

**FIGURE 5:**
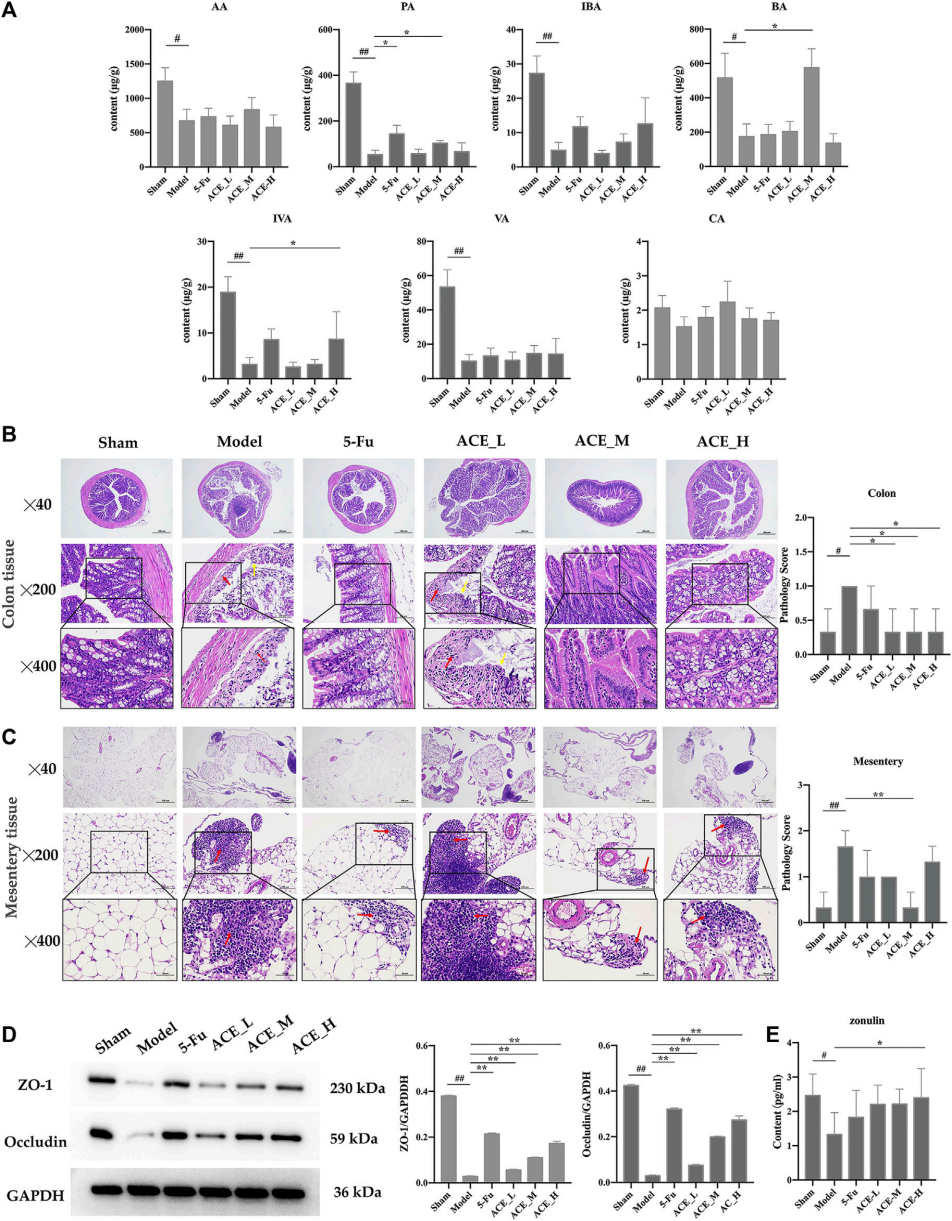
The effect of *Astragalus mongholicus* Bunge-*Curcuma aromatica* Salisb. extract on SCFAs and the intestinal barrier of colon cancer-bearing mice. **(A)** The contents of short-chain fatty acids in stool specimens after ACE administration. **(B)** Colon tissue histopathological changes (haematoxylin and eosin (HE) stain), disappearance of the mucosal layer (yellow arrows), and inflammatory cell infiltration (red arrows). **(C)** Conjunctival tissue histopathological changes (haematoxylin and eosin (HE) stain) and inflammatory cell infiltration (red arrows). **(D)** Expression of tight junction proteins (ZO-1 and Occludin) detected by WB. **(E)** The content of zonulin in serum detected by ELISA. ^#^ *p* < 0.05 and ^##^ *p* < 0.01, compared with the sham group; *p < 0.05 and **p < 0.01, compared with the model group. n = 10.

The contents of the seven SCFAs were determined in the intestinal contents of each group of mice (Figure 5A and Supplementary Table S2). Compared with the sham group, the contents of acetic acid, propionic acid, butyric acid, isobutyric acid, valeric acid, and isovaleric acid in the faeces of mice in the model group were reduced significantly (*p* < 0.05, *p* < 0.01), while the decrease in the content of hexanoic acid was not significant. After ACE administration, the faecal content of SCFAs was increased; specifically, the changes in the intestinal faecal propionic acid and butyric acid contents of mice in the colon cancer model were significantly reversed by ACE treatment (*p* < 0.05). This finding implies that the ameliorative effect of ACE on intestinal damage in colon cancer may be achieved by improving the content of SCFAs in the intestinal content of colon cancer-bearing mice.

As shown in Figure 5B, the mucosal layer of the intestinal glands in the model group partially disappeared (yellow arrows), and the surrounding intestinal glands were irregularly arranged with small areas of erosion and inflammatory cell infiltration (red arrows). In the 5-FU group, epithelial cells were also shed, and lymphocytes were infiltrated. The pathological changes in the mice in the ACE_L group were obvious, with epithelial cells detached from the mucosal layer, connective tissue hyperplasia and a small amount of inflammatory cell infiltration. In the mice in the sham-operated, ACE_M and ACE_H groups, the intestinal glands were closely arranged without obvious abnormalities. Severe focal infiltration of lymphocytes in the mesentery in tumour-bearing mice was also shown, and ACE showed a significant improvement after administration, especially in the mid-dose group (red arrows, Figure 5C).

Tight junction protein (TJP) is the main element in maintaining intestinal barrier function and is consistent with histomorphological changes in the colon. The protein expression levels of ZO-1 and occludin were significantly reduced in colon tissue after colon cancer modelling (*p* < 0.01, Figure 5D). The expression levels of ZO-1 and occludin were significantly upregulated after administration of different doses of ACE (*p* < 0.01), indicating that the intestinal mechanical barrier was repaired. High doses of ACE had a better influence than low and medium doses in increasing the protein expression levels of ZO-1 and occludin. Compared with the sham mice, the content of zonulin was decreased significantly in the serum of cancer-bearing mice (*p* < 0.05). After drug administration, the content was increased in the cancer-bearing mice; in particular, the high dose of ACE showed a significant upregulation (*p* < 0.05, Figure 5E).

### ACE Altered the SDF-1/CXCR4 Pathway to Adjust Inflammatory Factors Associated With Colon Cancer Progression

**FIGURE 6:**
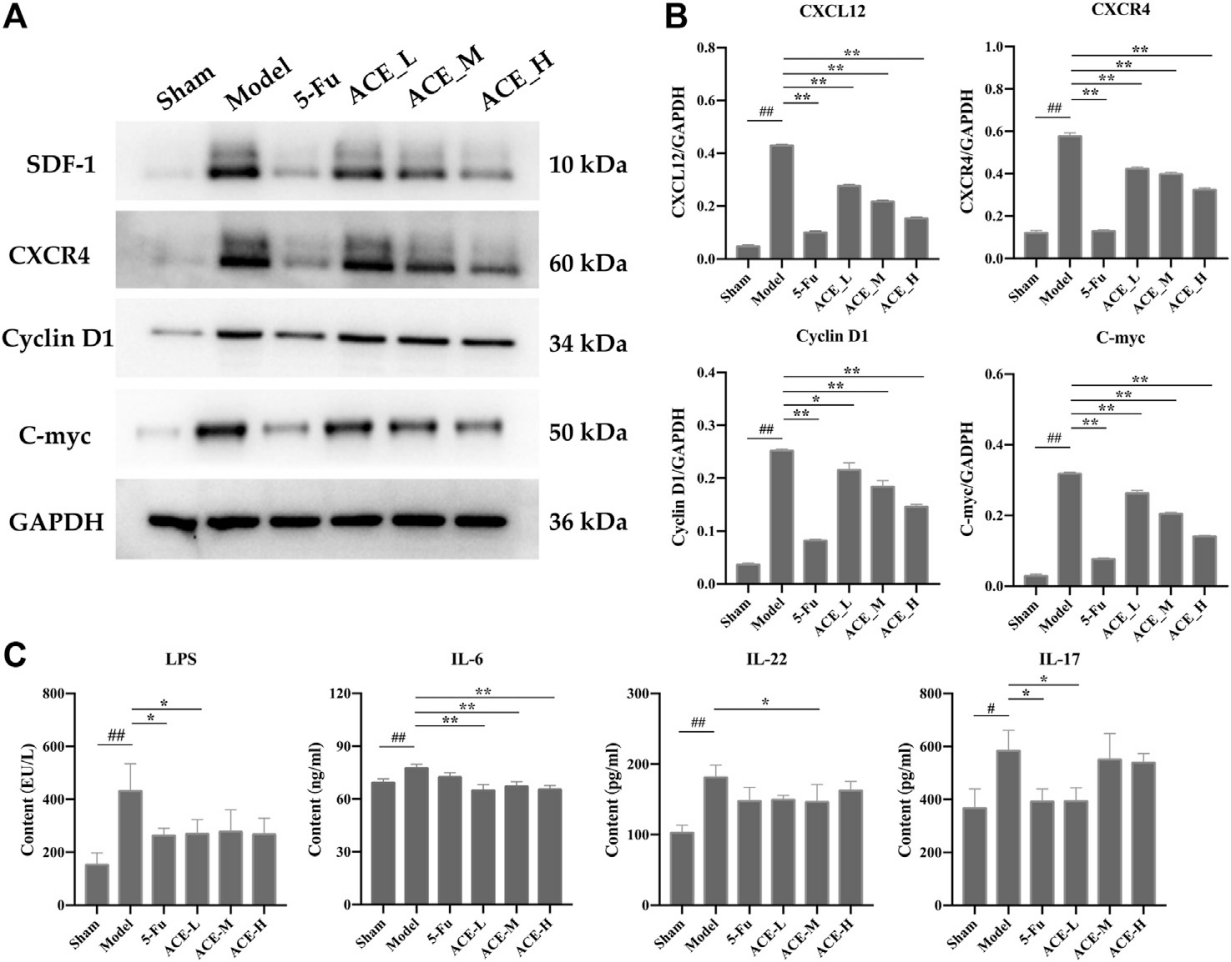
The effect of *Astragalus mongholicus* Bunge-*Curcuma aromatica* Salisb. extract on SDF-1/CXCR4 and inflammatory factors in colon cancer-bearing mice. **(A)** Greyscale strips of SDF-1, CXCR4, Cyclin D1 and C-myc detected by WB; **(B)** Protein expression of SDF-1, CXCR4, Cyclin D1 and C-myc; **(C)** The content of LPS, il-6, IL-22 and IL-17A in serum detected by ELISA. ^#^ *p* < 0.05 and ^##^ *p* < 0.01, compared with the sham group; *p < 0.05 and **p < 0.01, compared with the model group. n = 10.

The protein and mRNA expression levels of SDF-1 and CXCR4 and their downstream proteins cyclin D1 and c-myc in the tumour tissues of mice with colon cancer were detected by Western blot. The results in **Figure 6A, B** show that the protein expressions of SDF-1, CXCR4, cyclin D1 and c-myc in the tumour tissues of colon cancer mice were significantly upregulated (*p* < 0.01). However, the results in the ACE intervention groups were reversed, as the expression levels of SDF-1, CXCR4, cyclin D1 and c-myc were callback to those in the sham group (*p* < 0.05, *p* < 0.01). Similar to the differential microbiota results, systemic inflammatory cytokines were positively correlated with the colon cancer process, and key protein levels of LPS, IL-6, IL-22 and IL-17A in mouse serum were significantly higher after modelling than in the sham group (*p* < 0.01 or *p* < 0.01, Figure 6C). After ACE administration, the indicators of inflammation were relieved to some extent, especially the levels of IL-6 and IL-17A (*p* < 0.05).

## Discussion

Colon cancer, a malignant tumour that forms in colon tissue, causes the leading cancer-related deaths all over the world. At present, the main clinical treatment options are surgery and chemotherapy (Iveson et al., 2019). However, the therapeutic efficacy of anticancer agents has been limited due to the cancer stage and location at the period of diagnosis, the individual characteristics, as well as the drug resistance which develops in nearly all cancer patients (Gao et al., 2019). Therefore, it is a hot and urgent topic in modern medical research to find suitable, effective and safe anti-tumor drugs.

CHM has been demonstrated to exhibit antitumour effects in many cancers, and compared to radiotherapy and chemotherapy, it could enhance quality of life and prolong the survival rate with fewer toxic effects (Xiang et al., 2019). *Astragalus mongholicus* Bunge, a typical CHM used in many clinical antitumour formulas, contains saponins, flavonoids and polysaccharides and shows pivotal anticancer efficacy in multiple types of cancers (Wang et al., 2018; Li W. et al., 2019; Wang et al., 2021). *Curcuma aromatica* Salisb. mainly contains essential oil components and is frequently used in the clinic for tumour treatment (Liu et al., 2019). Its main bioactive compounds, such as β-elemene, curcumol, and curcumin, also display significant anticancer activity by inducing apoptosis, targeting miRNA, and modulating gut microbiota (Di Meo et al., 2019; Giordano and Tommonaro, 2019). The results of UPLC–MS/MS analysis showed that ACE contained astragaloside I, astragaloside II, astragaloside A, formononetin, ononin, calycosin, calycosin-7-glucoside, β-elemene, curzerene, curdione, germacrone, curcumin, demethoxycurcumin and bisdemethoxycurcumin. Among them, astragaloside A (astragaloside IV) is regarded as a standard of content determination for quality control of *Astragalus mongholicus* Bunge, can inhibit cancer progression and metastasis (Jia et al., 2019), enhance cisplatin chemosensitivity in cancer, modulate gut microbiota profile and promote butyric acid generation (He et al., 2020). β-elemene, one of the main ingredients in essential oils of *Curcuma aromatica* Salisb., has been widely used as in clinical anti-tumor treatment as elemene injection (Zhai et al., 2020). In addition, previous studies have shown that curcumin and germacrone derived from *Curcuma aromatica* Salisb. effctively suppressed the development of tumor by improving intestinal barrier function, modulating colonic microbiota and promoting autophagosomes formation (Giordano and Tommonaro, 2019; Zhao et al., 2020). Consistent with the results of previous studies, the combination of *Astragalus mongholicus* Bunge and *Curcuma aromatica* Salisb. (ACE) significantly inhibited the growth and metastasis of colon cancer and improved pathological histological changes in solid tumours and liver tissue.

Numerous data support that altered microbiota communities can modulate the efficacy of anticancer drugs via mediating or modifying the environmental factors of colon cancer (Nakatsu et al., 2015; Mori and Pasca, 2021). The proportional imbalance between pathogenic and beneficial bacteria aggravates intestinal mucositis. Analysis of the mucosa faeces of patients undergoing cancer therapy shown that bateria that ferment fibre and produce short-chain fatty acids (SCFAs) are siginificantly decreased. In our study, the abundance and diversity of the gut microbiota was significantly reduced in mice after colon cancer modelling, and the flora structure was restored after ACE administration, while 5-FU showed no significant improvement in this parameter. *Lactobacillus* is a common probiotic with health maintenance and immune modulating properties, and its increased abundance inhibits the multiplication of pathogenic bacteria (Sharma et al., 2014). *Roseburia* is capable of producing butyrate, which in moderate amounts can relieve inflammation to maintain a healthy gut (Kellermayer, 2019). The genus *Mucispirillum*, a mucosal-resident bacterium, can form a colony film in which inherently mucosa-resident bacteria can protect the host by inhibiting contact between the pathogen and the host mucosa. The increased proportions of *Akkermansia muciniphila* and *Mucispirillum schaedleri* positively regulated the thickness of the intestinal mucus layer and the integrity of the intestinal barrier and promoted the effectiveness of immunotherapy. *Mucispirillum* also showed a significant negative correlation with TNF-α (Campbell et al., 2018; Alvarado et al., 2019). Pathogenic bacteria, such as *Escherichia*-*Shigella* and *Streptococcus* and *Enterococcus*, were significantly increased in CRC in the intestinal microbiota. Therefore, damage to the intestinal barrier was increased due to the increasing pathogenic factors (endotoxins and ammonia) produced by these bacteria (Han S. et al., 2019). Some harmful bacteria, such as *Escherichia*-*Shigella,* can still increase significantly after 5-FU induction (Chen et al., 2020). Our present study showed that ACE treatment significantly attenuated intestinal microbial dysbiosis in colon cancer-bearing mice. ACE increased the levels of probiotic gut microbiota like *Prevotellaceae_UCG-001*, *Lactobacillus* and *Roseburia*, whereas inhibited the levels of pathogenic gut bacteria, including *Escherichia-Shigella*, *Streptococcus* and *Enterococcus*.

With the probiotic bacteria decreasing in colon cancer, SCFAs produced by *Lactobacillus*, *Prevotellaceaee_UCG-001* and *Roseburia,* were also decreased. As one of the important metabolites of intestinal microbiota, SCFA supplementation enhances IL-22 production, which protects intestines from inflammation (Yang et al., 2020). They affect a series of activities of the host and play a key effect on maintaining energy homeostasis and intestinal barrier integrity and have some clinical implications in the treatment of disease. It was reportwd that after four SCFA-producers (*Bifidobacterium longum, Clostridium symbiosum, Faecalibacterium prausnitzii,* and *Lactobacillus fermentum*) transplantation, poststroke neurological deficits and inflammation were alleviated, and gut, brain and plasma SCFA concentrations were elevated in aged stroke mice (Lee et al., 2020). Overall changes in anaerobic bacteria can be assessed by measuring the levels of SCFAs, and their levels may also reflect bacterial activity. SCFAs are involved in the development and progression of colon cancer by regulating gastrointestinal metabolism (Albasri et al., 2019). Our results indicated that the content of SCFAs in the intestine of colon cancer-bearing mice was significantly decreased compared with the sham mice, except for hexanoic acid. Among these, acetic acid, propionic acid and butyric acid produced by the degradation of carbohydrates via intestinal bacteria accounted for most of the contents (Li L. et al., 2019), and regulate the absorption of various nutrients and hormone production in the intestine, and are widely involved in energy metabolism. Lower level of butyrate-producing bacteria was observed in the gut of people with metabolic and inflammatory diseases (Kasahara et al., 2018). And butyric acid can increase the production of *Lactobacillus* and reduce the number of *Escherichia coli*, which means it stores energy (Patman, 2015). The content of SCFAs increased to varying degrees after ACE administration, but only the levels of propionate and butyrate were reversed significantly in colon cancer-bearing mice at ACE medium doses.

Dysbiosis of the intestinal flora can lead to alterations in key proteases and metabolites that induce the degradation of E-calmodulin, leading to dysfunction of barrier permeability and activation of Wnt/β-catenin and NF-κB signalling pathways that promote colon cancer development (Elinav et al., 2013). Furthermore, chemokines produced by colon cancer cells could be stimulate by the intestinal microbiota. SDF-1, the only ligand of CXCR4, is widely expressed at the apical surface of human intestinal epithelial cells (Teicher and Fricker, 2010). It was shown that butyrate as a histone deacetylase inhibitor--HDACi and other SCFAs significantly suppressed the expression of SDF-1, and sodium butyrate also up-regulated CXCR4 expression (Gupta et al., 2001; Vangaveti et al., 2014; Chandra Roy et al., 2018). SDF-1 and CXCR4 are commonly highly expressed in various human malignancies (Xu et al., 2015), and the SDF-1/CXCR4 axis plays a central role in the development of cancer. The SDF-1/CXCR4 axis has been demonstrated to activate the transcription factor NF-κB, which affects the proliferation and survival of cancer cells. We also examined the protein and mRNA expression levels of the anti-colon cancer action pathway, SDF-1/CXCR4 axis before and after intervention with the ACE drug pair. The results showed that ACE treatment could significantly reduce both the protein and mRNA expression levels of SDF-1 and CXCR4 in tumour tissues.

There might be a crucial function for SDF-1/CXCR4 in promoting chemotaxis lung cancer cells gather to the brain astrocytes and secreting lysozyme to damage BBB. AMD3100, CXCR4 antagonist, exerts therapeutic effects on experimental colitis by inhibiting colonic inflammation and enhancing epithelial barrier integrity (Xia et al., 2010). The activation of CXCR4 regulates mucosal host defense through stimulating epithelial cell migration and promoting intestinal barrier integrity (Agle et al., 2010). Intestinal tight junction proteins (TJPs) regulate the permeability of the intestinal barrier. Zonula occludens-1 (ZO-1) and occludin are essential in maintaining intestinal barrier function. Studies have shown that the protein expressions of ZO-1 and occludin are reduced in adenocarcinoma of the digestive tract and that the intestinal barrier is impaired, followed by increased intestinal permeability (Han X. et al., 2019). Simultaneously, butyrate in colon contents could decrease expressions of occludin-1 and ZO-1 in colon tissue (Wei et al., 2020). In the present study, intestinal barrier was injured after colon cancer modelling, accompanied with infiltration of lymphocytes, monocytes in the intestinal mucosal layer, and a decreasing in intestinal tight junction proteins. After ACE intervention, the alteration was reversed siginificantly compared with those in the tumour-bearing mice.

Cell cycle regulatory protein Cyclin D1 and C-myc gene expressions could be stimulated by CXCR4 and its ligand CXCL12 (Dimova et al., 2014). Cyclin D1 is a cell cycle regulatory protein that drives the transition from G1 to S phase of the cell cycle and promotes cell proliferation. Many studies have found that Cyclin D1 is highly expressed in colon cancer and is considered to be an independent factor for poor prognosis, and it could be inhibited by inhibiting NF-κB (Bimonte et al., 2015). The oncogene C-myc, the endogenous homologue of the oncogene V-myc, has been shown to have a transforming effect and has a dual function of regulating cell proliferation and apoptosis. It also facilitates the transition of cells to a malignant phenotype, is associated with tumour development, metastasis and is also associated with patient prognosis (Al-Kuraya et al., 2007). We also detected the protein and mRNA expression levels of the downstream molecules of SDF-1/CXCR4 axis before and after intervention with the ACE drug pair. The results showed that ACE treatment could significantly reduce expressions of Cyclin D1 and C-myc of tumour tissues both in protein and mRNA level.

The dose levels used here followed the dosages tested in this study were based on the body surface area conversion, which, however, resulted in a dose level which is likely to cause artefacts in the model used. Therefore, studies at a therapeutically more relevant dose level are suggested as one of the next steps.

## Conclusion

**FIGURE 7:**
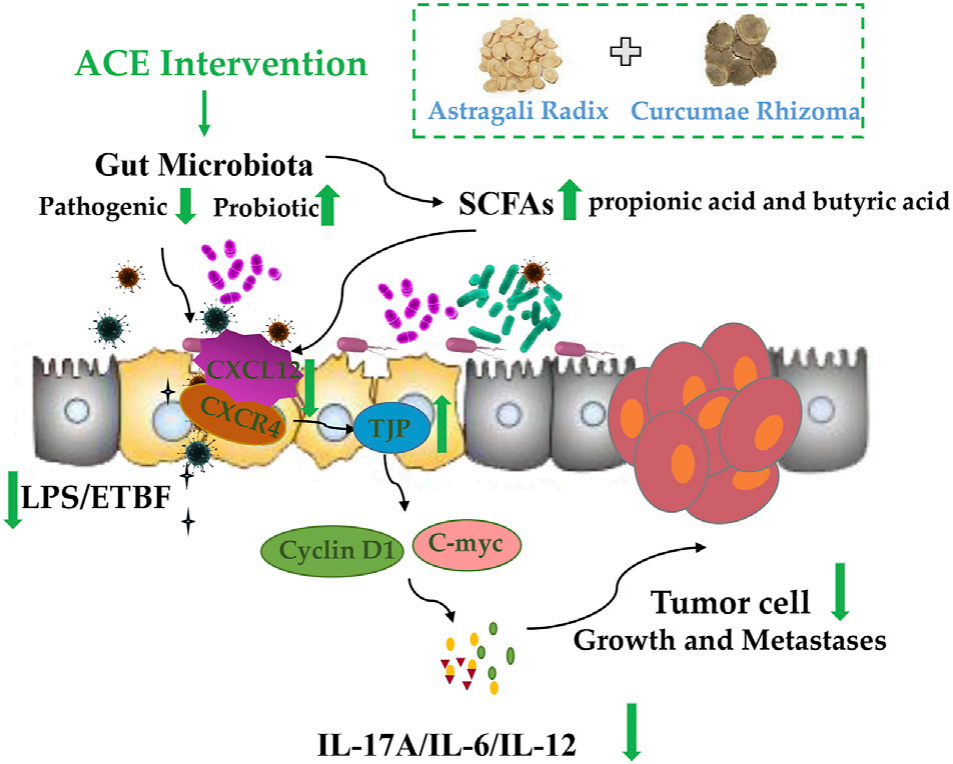
Standardized *Astragalus mongholicus* Bunge-*Curcuma aromatica* Salisb. extract efficiently suppresses colon cancer progression through gut microbiota modification in CT26-bearing mice.

Overall, our findings highlight a real need for a more complete understanding of how *Astragalus mongholicus* Bunge-*Curcuma aromatica* Salisb. (ACE) influences the progression of colon cancer via the gut microbiome in CT26-bearing mice. The mechanism of colon cancer progression is relate to intestinal bacteria dysbiosis, and the drug pair ACE might improve the diversity and abundance of the related gut microbiota, in turn to increase the content level of SCFAs such as propionic acid and butyric acid, which further mediate intestinal SDF-1/CXCR4 signalling pathway to repair the integrity of the intestinal barrier, decrease Cyclin D1 and C-myc expressions, eventually inhibiting the growth and metastasis of colon cancer (Figure 7).

## Data Availability

The data generated in this article can be found in NCBI using accession number PRJNA738850.
