# Supplementary material for: Standardized Astragalus Mongholicus Bunge-Curcuma Aromatica Salisb. Extract Efficiently Suppresses Colon Cancer Progression Through Gut Microbiota Modification in CT26-Bearing Mice
Source: Front Pharmacol. 2021 Aug 31;12:714322. doi: 10.3389/fphar.2021.714322 (PMC8438123; doi:10.3389/fphar.2021.714322)
Supplement: Supplementary file 1 [file DataSheet1.docx]

***Supplementary Material***

**1. Method validation of UPLC–MS/MS**

- 1. *Calibration curves, limits of detection and quantification*

The standard solution was diluted to prepare a series of appropriate concentrations for the establishment of calibration curves. Calibration curves were constructed from peak areas versus their corresponding concentrations of the reference standards. The results are shown in Table S1. The r^2^ values were all above 0.990, indicating good linearity of the components to be tested. The lower limit of detection (LOD) and the lower limit of quantification (LOQ) were determined at signal-to-noise ratios (S/N) of 3 and 10, respectively.

- 1. *Precision*

Medium concentrations of the standards were injected 6 times in succession to evaluate the precision. The RSD of each component to be measured was ≤ 1.93% (shown in Table S1), indicating that the precision of the instrument was good.

- 1. *Repeatability*

Six independent ACE sample solutions were prepared and analysed to measure repeatability. The results showed that the RSD of each component to be tested was ≤ 1.99% (shown in Table S1), indicating that the method was reproducible.

- 1. *Stability*

For stability evaluation, one of the mixed standard solutions stored at 20 °C was analysed at 0, 2, 4, 8, 12, 24 and 48 h. The results showed an RSD ≤ 1.88% for each of the components to be measured (shown in Table S1), indicating that the stability of the test solution was good over 48 h.

**2. Total ion flow chromatogram and detection methodology of SCFAs**

- 1. *Total ion flow chromatogram*

As shown in the TIC diagram (Figure S1), all short-chain fatty acids can be distinguished. The internal standard (isocaproic acid) peaked at 9.53 min and was clearly separated from the other standards, indicating a good method.

- 1. *Linear range of the standard curve*

The calibration curve was constructed using the concentration of the standard as the horizontal coordinate and the ratio of the peak area of the standard to the internal standard as the vertical coordinate to obtain the linear regression equation (r^2^>0.990) (Table S2).

- 1. *Precision*

The mixed standard at a concentration of 25 μg/mL was injected 8 times in succession to calculate the intraday precision and processed on days 1, 2 and 3 to determine the interday precision. The results are expressed as the RSD. The intraday precision ranged from 1.00% to 1.85%, and the interday precision ranged from 4.76% to 11.73%, indicating that the instrument was of good precision (Table S2).

- 1. *Repeatability*

Six samples were processed repeatedly to calculate the repeatability, and the results are expressed as RSDs (Table S2).

- 1. *Recovery rate*

QC samples at low, medium and high concentrations (LQC, MQC, and HQC) processed 6 copies in parallel, and the recovery was measured on the same day. Since each target substance is endogenous, recovery= (actual value - theoretical value)/amount added*100% (Table S2).

The results showed good linearity of the substances, intraday and interday precision and repeatability were less than 15%, and the recoveries were between 80% and 115%, which met the analytical requirements, indicating that the method is stable, reliable and can be applied to determine our samples.

**3. Assessment of the gut microbiome**

As shown by the species dilution curve (rarefaction curve)-Shannon index plot, the curves for each sample tended to flatten out, indicating that the sequencing data reached saturation and were able to cover the majority of species in the mouse gut microbiome community (Figure S1).

**
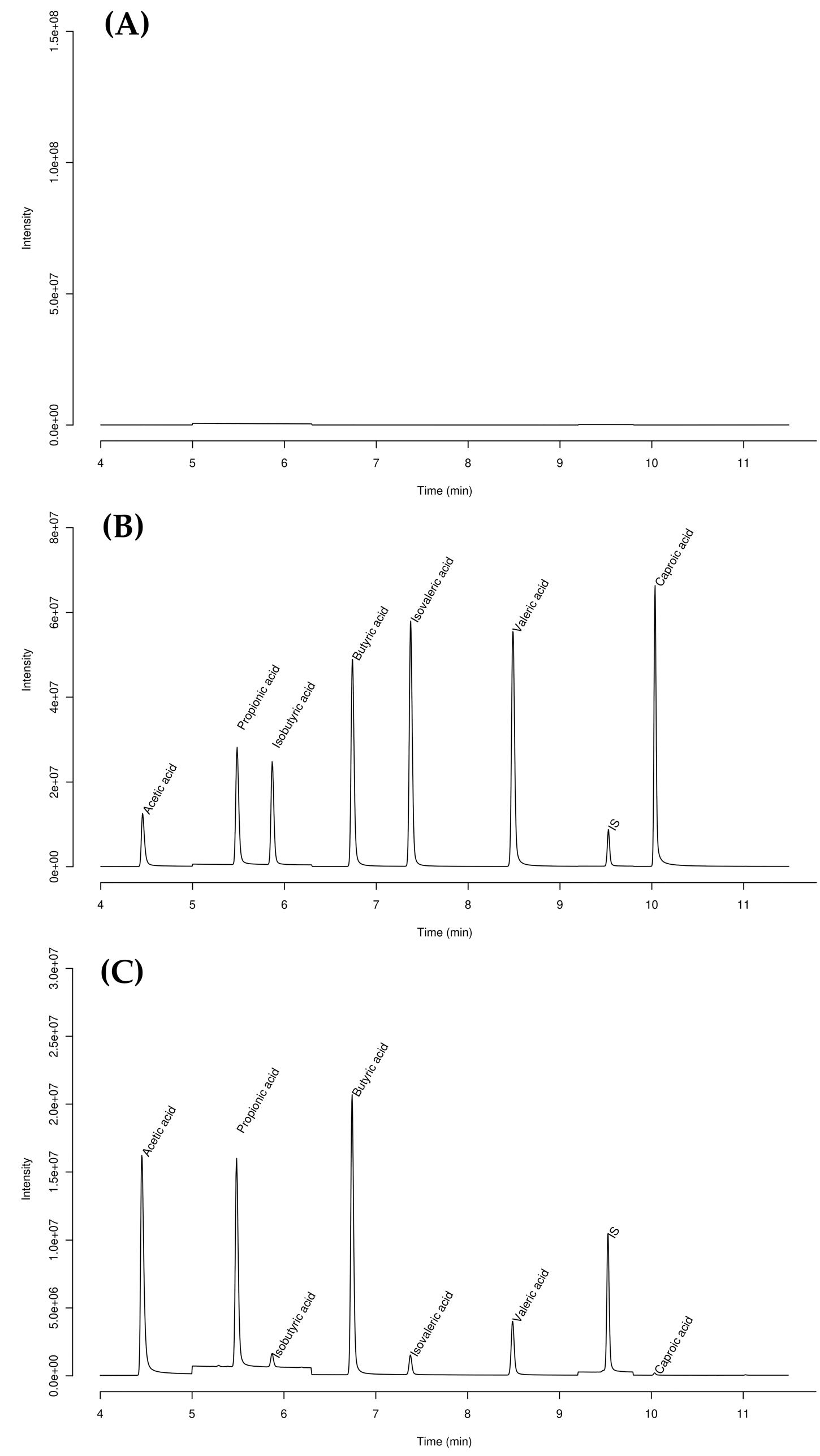
**

**Figure S1.** Total ion chromatogram (n=6) A. Blank; B. Mixed Standards; C. Samples


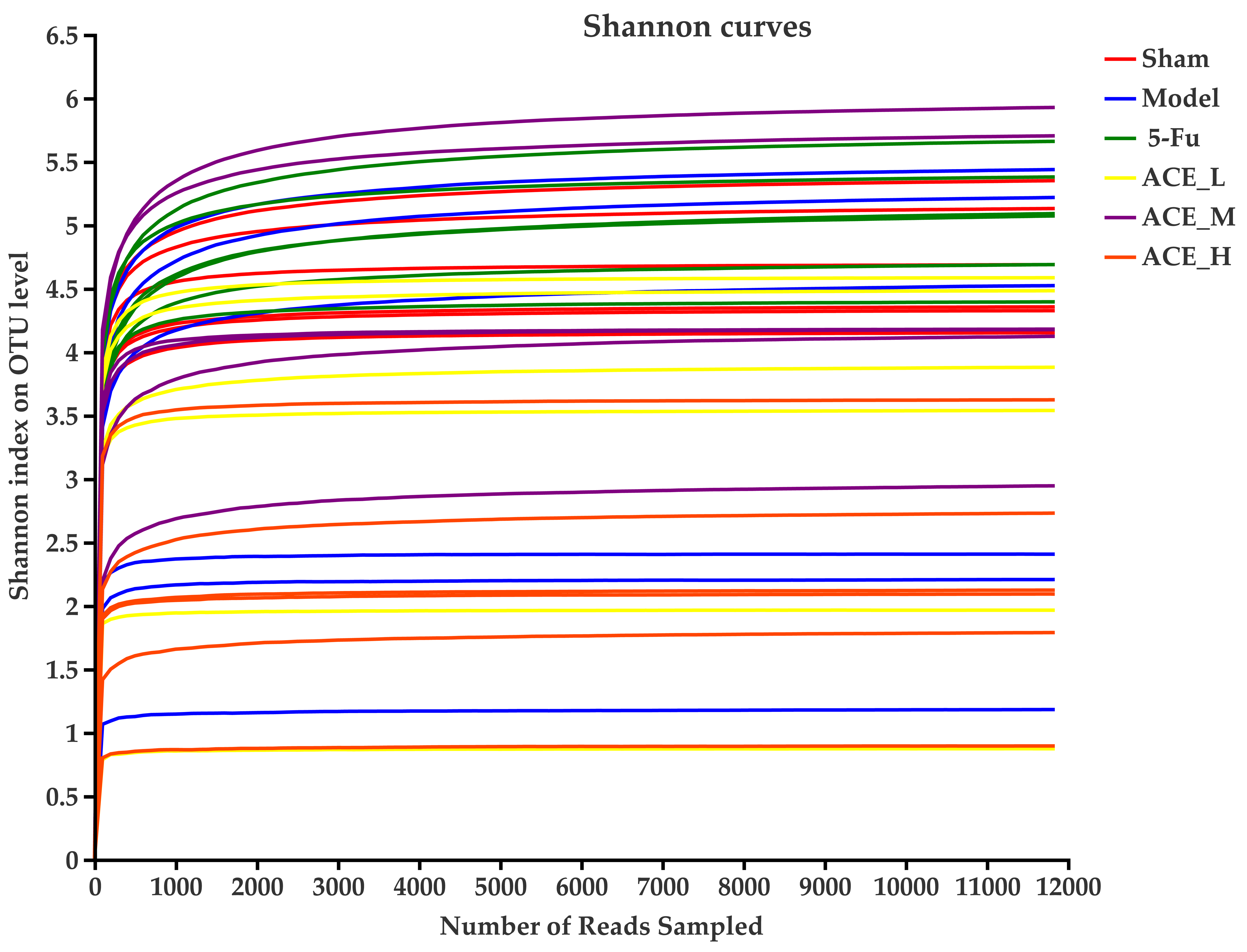


**Figure S2.** Shannon curves of all samples


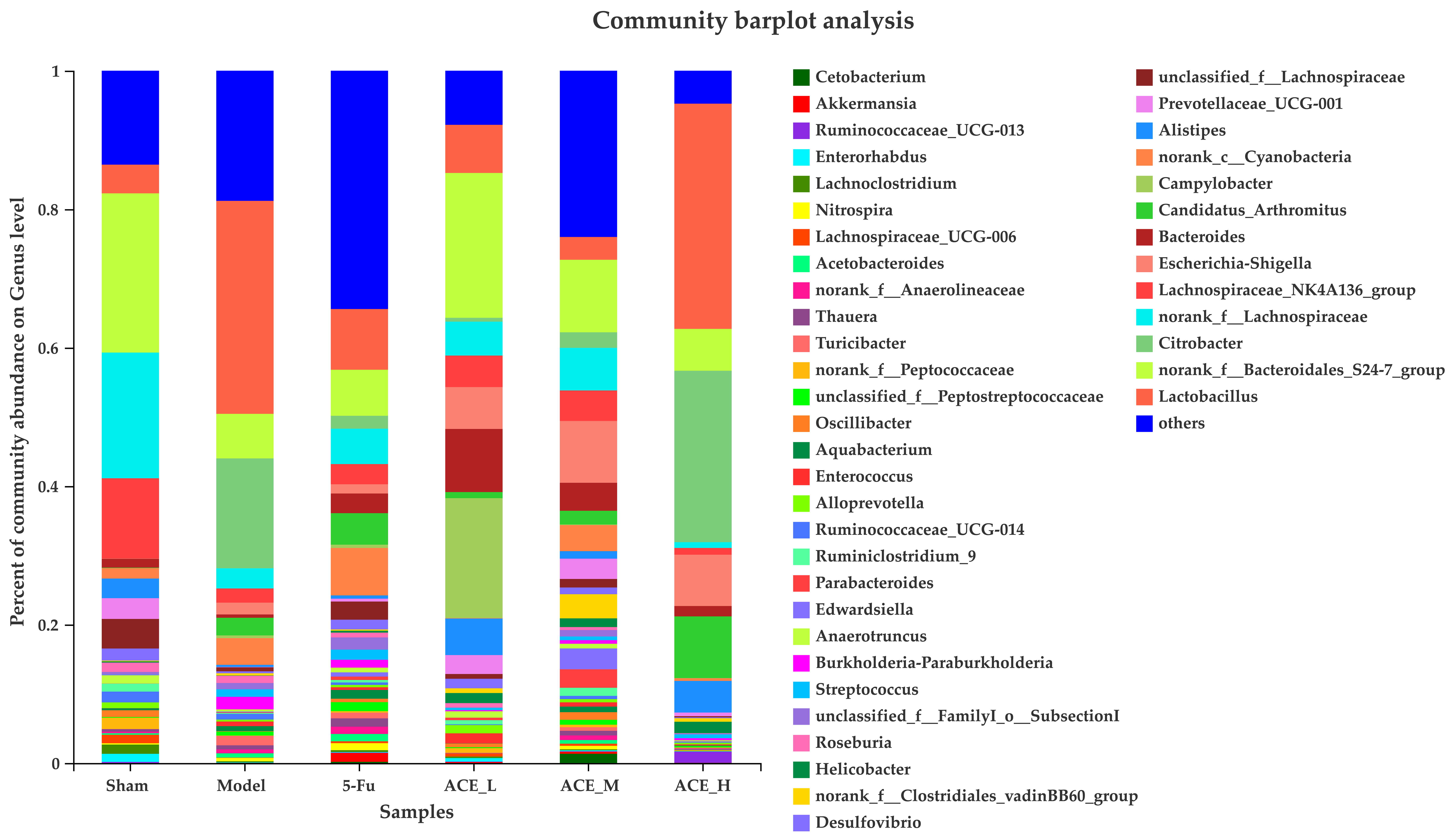


**Figure S3.** Community bar plot analysis of gut microbiota at the genus level (n=6)


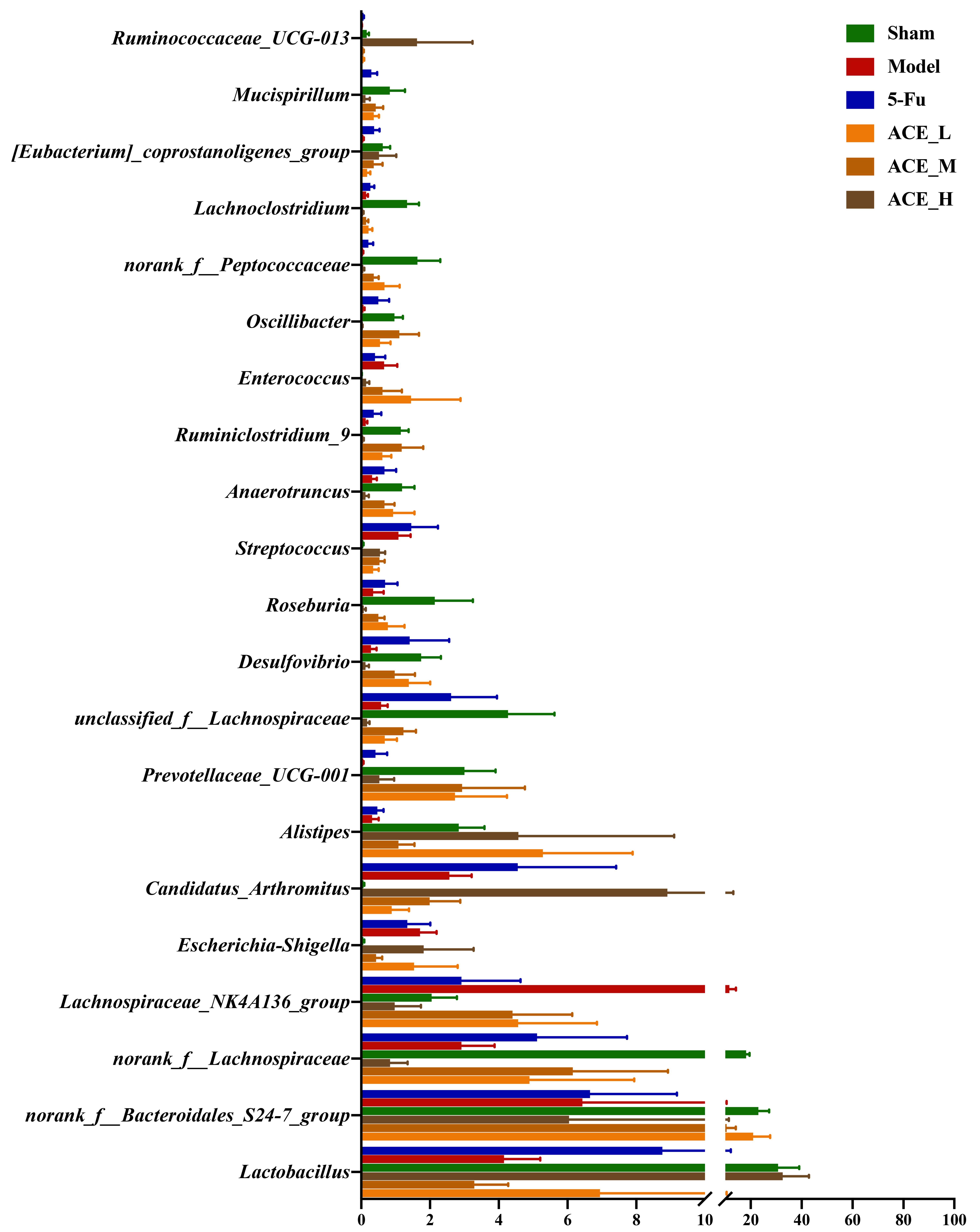


**Figure S4.** Twenty-one top differentially regulated genera (n=6)

**Table S1.** Regression equations, linear ranges and the methodological study of UPLC–MS/MS

| **NO.** | **Standards** | **Calibration curves** | **r^2^** | **Linear range (μg/mL)** | **LOD**  **(ng/mL)** | **LOQ**  **(ng/mL)** | **RSD/%** | | |
| --- | --- | --- | --- | --- | --- | --- | --- | --- | --- |
|  |  |  |  |  |  |  | **Precision**  **(n=6)** | **Repeatability (n=6)** | **Stability (n=6)** |
| 1 | calycosin-7-glucoside | y = 15,254,543.14 x + 829,700.41 | 0.991 | 0.012~1.533 | 3.636 | 11.977 | 1.79 | 1.92 | 0.61 |
| 2 | ononin | y = 3,413,745.18 x + 128,577.96 | 0.992 | 0.011~0.730 | 3.333 | 11.403 | 1.40 | 1.47 | 0.82 |
| 3 | calycosin | y = 206190x + 17113 | 0.992 | 0.012~2.971 | 3.636 | 12.145 | 1.89 | 1.47 | 1.89 |
| 4 | astragaloside A | y = 8297.3x + 576.82 | 0.997 | 0.009~4.632 | 2.727 | 9.048 | 0.86 | 1.55 | 0.28 |
| 5 | astragaloside II | y = 613212x + 95478 | 0.996 | 0.014~14.324 | 4.242 | 13.988 | 1.61 | 1.14 | 1.88 |
| 6 | formononetin | y = 10,571,285.56 x + 131,071.51 | 0.994 | 0.012~0.194 | 3.636 | 12.150 | 1.85 | 1.58 | 0.51 |
| 7 | astragaloside I | y = 970737x + 109825 | 0.997 | 0.009~9.235 | 2.727 | 9.019 | 1.64 | 1.85 | 1.74 |
| 8 | bisdemethoxycurcumin | y = 29,096,512.44 x + 4,029,216.05 | 0.991 | 0.029~3.702 | 8.788 | 28.924 | 1.80 | 4.84 | 0.11 |
| 9 | demethoxycurcumin | y = 33,705,904.35 x + 1,380,475.50 | 0.997 | 0.015~1.858 | 4.545 | 14.519 | 1.42 | 1.63 | 1.72 |
| 10 | curcumin | y = 17,343,459.01 x + 1,227,929.63 | 0.996 | 0.014~3.710 | 4.242 | 14.491 | 1.85 | 1.91 | 1.61 |
| 11 | curdione | y = 34,785,375.41 x + 1,657,959.99 | 0.996 | 0.014~1.825 | 4.242 | 14.261 | 0.61 | 1.90 | 1.52 |
| 12 | curzerene | y = 61937x + 80961 | 0.996 | 0.022~91.294 | 6.667 | 22.289 | 1.31 | 1.80 | 1.27 |
| 13 | germacrone | y = 2,085,534.13 x + 436,786.57 | 0.994 | 0.014~14.574 | 4.242 | 14.232 | 1.93 | 1.99 | 1.51 |
| 14 | β-elemene | y = 321568x + 249792 | 0.999 | 0.015~59.941 | 4.545 | 14.634 | 1.29 | 1.92 | 0.76 |

**Table S2.** The linear regression equation, precision, repeatability and limit of quantification of standard products (n=6)

| **Standards** | **Calibration curves** | **r^2^** | **Linear range (μg/mL)** | **RSD (%)** | | | **LOQ**  **(μg/mL)** | **Recovery rate (%)** | | |
| --- | --- | --- | --- | --- | --- | --- | --- | --- | --- | --- |
|  |  |  |  | **Intra-day Precision** | **Inter-day Precision** | **Repeatability** |  | **LQC**  **(μg/mL)** | **MQC**  **(μg/mL)** | **HQC**  **(μg/mL)** |
| acetic acid | y=0.019x + 0.0033 | 0.9961 | 0.02-500 | 1.85 | 11.73 | 5.44 | 0.02 | 90.34 | 97.25 | 106.31 |
| propionic acid | y=0.0115x + 0.0003 | 0.9973 | 0.02-500 | 1.73 | 5.28 | 4.72 | 0.02 | 94.55 | 87.81 | 87.53 |
| isobutyric acid | y=0.0166x + 0.0001 | 0.9978 | 0.02-500 | 1.31 | 4.76 | 5.65 | 0.02 | 207.07 | 86.17 | 85.2 |
| butyric acid | y=0.0709x + 0.0023 | 0.9948 | 0.02-500 | 1.57 | 6.87 | 5.51 | 0.02 | 96.55 | 85.56 | 86.38 |
| isovaleric acid | y=0.0825x + 0.0005 | 0.9919 | 0.02-500 | 1.35 | 6.63 | 7.59 | 0.02 | 92.82 | 85.50 | 85.8 |
| valeric acid | y=0.0812x + 0.0002 | 0.9962 | 0.02-500 | 1.3 | 5.66 | 7.68 | 0.02 | 99.25 | 88.16 | 89.19 |
| hexanoic acid | y=0.0699x + 0.0012 | 0.9958 | 0.02-500 | 1 | 7.71 | 9.53 | 0.02 | 85.22 | 96.97 | 96.76 |
